# Chemical Compounds and Ambient Factors Affecting Pancreatic Alpha-Cells Mass and Function: What Evidence?

**DOI:** 10.3390/ijerph192416489

**Published:** 2022-12-08

**Authors:** Gaia Chiara Mannino, Elettra Mancuso, Stefano Sbrignadello, Micaela Morettini, Francesco Andreozzi, Andrea Tura

**Affiliations:** 1 Department of Medical and Surgical Sciences, Magna Graecia University of Catanzaro, 88100 Catanzaro, Italy; 2 CNR Institute of Neuroscience, 35127 Padova, Italy; 3 Department of Information Engineering, Università Politecnica delle Marche, 60131 Ancona, Italy

**Keywords:** environment, alpha cell, glucagon, air pollutants, toxic chemicals, pharmaceutical agents, fatty acids, heat exposure

## Abstract

The exposure to different substances present in the environment can affect the ability of the human body to maintain glucose homeostasis. Some review studies summarized the current evidence about the relationships between environment and insulin resistance or beta-cell dysfunction. Instead, no reviews focused on the relationships between the environment and the alpha cell, although in recent years clear indications have emerged for the pivotal role of the alpha cell in glucose regulation. Thus, the aim of this review was to analyze the studies about the effects of chemical, biological, and physical environmental factors on the alpha cell. Notably, we found studies focusing on the effects of different categories of compounds, including air pollutants, compounds of known toxicity present in common objects, pharmacological agents, and compounds possibly present in food, plus studies on the effects of physical factors (mainly heat exposure). However, the overall number of relevant studies was limited, especially when compared to studies related to the environment and insulin sensitivity or beta-cell function. In our opinion, this was likely due to the underestimation of the alpha-cell role in glucose homeostasis, but since such a role has recently emerged with increasing strength, we expect several new studies about the environment and alpha-cell in the near future.

## 1. Introduction

It is well known that the exposure to different substances present in the environment can affect the ability of the human body to maintain glucose homeostasis, thus possibly contributing to the onset of diabetes or to the worsening of the metabolic condition in people already suffering from the disease. In recent years, several review and meta-analysis studies have summarized the scientific knowledge on the issue, focusing specifically on the effect of several environmental factors and compounds in the most common types of diabetes, i.e., type 1 diabetes [1,2,3,4,5,6,7,8,9,10,11,12,13,14,15], type 2 diabetes [16,17,18,19,20,21,22,23,24,25,26,27,28,29,30,31,32,33,34,35,36], and gestational diabetes [37,38,39,40,41,42,43,44,45,46,47]. In addition, several studies went into more detail about the relationships between environment and diabetes, focusing on specific etiological factors of diabetes. Specifically, some studies focused on the relationships between environment and insulin resistance [48,49,50,51,52,53,54,55,56,57,58,59,60,61]. Other studies mainly analyzed the relationships between environment and beta-cell dysfunction, with consequent impairment of insulin secretion [62,63,64,65,66,67,68,69,70,71,72].

The interest in the environment and insulin resistance or beta-cell dysfunction is conceivable and expected, since insulin sensitivity and beta-cell function are likely the most relevant factors in the regulation of glucose homeostasis. However, it is currently clearly established that other physiological factors play a remarkable role in glucose homeostasis. In a popular article by De Fronzo [73], eight factors were indicated as relevant for glucose homeostasis: in addition to decreased glucose uptake by muscles (insulin resistance) and decreased insulin secretion (beta-cell dysfunction), other relevant factors were identified in increased glucagon secretion (alpha-cell dysregulation), increased hepatic glucose production, increased lipolysis, increased glucose reabsorption, decreased incretin effect, and neurotransmitter dysfunction.

Among the indicated factors [73], the role of the alpha cell is interesting for its heterogeneous physiological mechanisms of action, not limited to the stimulation of hepatic glucose production through glucagon secretion but also participating in beta-cell function regulation [74]. However, to our knowledge, no review study has focused on the relationships between environment and alpha-cell function. The aim of this review is therefore to identify the relevant studies in the field, and summarize the evidence regarding the possible effects of both chemical/biological and physical environmental factors on the alpha cell.

## 2. Scientific Literature Search Strategy

A search of the scientific literature was performed in PubMed by one of the study authors, then checked and agreed upon by another author.

Following testing of different PubMed search strings, we identified this final string:

((alpha-cell*[ti] OR α-cell*[ti] OR glucagon*[ti]) AND (environment*[ti] OR atmospher*[ti] OR air[ti] OR pollut*[ti] OR contamina*[ti] OR ambien*[ti] OR habitat*[ti] OR expos*[ti] OR toxic*[ti] OR territor*[ti])) NOT glucagon-like*[ti].

According to PubMed guidelines, “ti” searches in the article title. The symbol “*” allows searching for all possible variations of a word root. Notably, we used different terms for our search, which are sometimes used interchangeably, though strictly speaking they are not.

**Figure 1:**
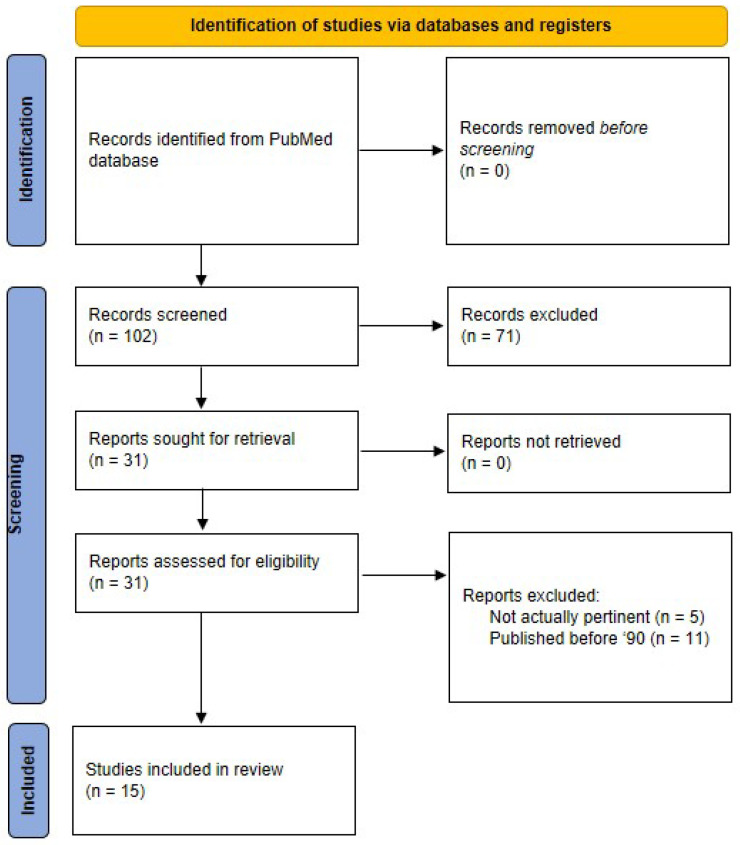
PRISMA flow diagram of the literature search strategy.

The indicated search strategy yielded 102 items (last check: 30 September 2022). We therefore analyzed each item and selected 26 articles as pertinent for our analysis (none of which was a review study). However, we noted that almost half of those articles were published several years ago. For this review, we decided to focus on the articles published in the nineties or later (i.e., the 1990–today range), ending up with a set of 15 articles. Nonetheless, we will briefly mention the elderly articles in the discussion. The PRISMA flow chart of our literature search is reported in Figure 1. In the following sections, we summarize the main aspects of the selected 15 studies. Articles are presented in two separate sections: the first section is related to studies concerning the effects on the alpha cell of chemical or biological compounds available in the environment, whereas the second section describes studies related to the effects on the alpha cell of physical environmental factors. The first section is then divided into two subsections. In each section or subsection, articles are reported in chronological order. Synthetic information about each study is reported in Table 1, Table 2 and Table 3.

## 3. Chemical and Biological Compounds Effect on the Alpha Cell

### 3.1. Various Chemical/Biological Compounds

In 2018, the study by Stošić et al. [75] aimed at examining the effects of acrylamide on the structural changes of pancreatic alpha cells (as well as beta cells). Indeed, acrylamide is a toxic compound used to synthesize polymers for industrial and laboratory processes and is also formed during the high-temperature preparation of foods rich in carbohydrates (such as in frying, roasting, and baking). Thus, common foods, such as bread, baked potatoes, coffee, cereals, and various confectionery products, contain acrylamide, implying that acrylamide is often ingested on a daily basis. In this study, a group of 20 adult male Wistar rats were orally treated with acrylamide at 25 or 50 mg/kg of body weight (10 rats for each group) for three weeks, while a third group of 10 rats was the control and received distilled water in the same way. Immunohistochemical evaluation of glucagon (and insulin) expression and stereological analyses of pancreatic alpha cells (and beta cells) were performed. Specifically, assessed stereological parameters of alpha and beta cells were volume density, numerical density, surface density, nuclear and cytoplasmatic volume density, and nucleocytoplasmic ratio. It was found that alpha cells in pancreatic sections showed a dose-dependent increase of surface, numerical, and volume densities in acrylamide-treated groups as compared to the control. Additionally, a dose-dependent increase in the volume density of alpha cell nuclei was observed in the treated groups, while the volume density of alpha cell cytoplasm did not show significant changes, resulting in an increase in the nucleocytoplasmic ratio of alpha cells. It was concluded that sub-chronic acrylamide treatment of adult rats leads to islet of Langerhans remodeling determined by alpha-cell expansion, with a consequent decline in beta-cell mass.

The study by Fang et al. in 2020 [76] was interested in the effects of environmental pollutants on the alpha cell (and beta cell as well). Specifically, the focus was on Aroclor 1254, which is a polychlorinated biphenyl mixture. To this purpose, 40 male mice were randomly divided into five groups and orally gavaged with Aroclor 1254 at different doses (0.5, 5, 50, and 500 μg/kg) every 3 days for 60 days, or with an equal volume of vehicle (5 μL/g) with no added Aroclor 1254 in the case of the control group. The pancreas’ histological examination showed that the degree of apoptosis was increased in a dose-dependent manner in both alpha cells and beta cells. In addition, the proliferation rate of the alpha cells was not affected by the Aroclor 1254 exposure, whereas that of the beta cells was increased. Furthermore, the alpha cells showed markedly decreased neogenesis, whereas again beta cells showed the opposite behavior. Overall, these physiologic phenomena triggered by the Aroclor 1254 exposure resulted in a decreased alpha-cell mass but an increased beta-cell mass. It was concluded that the beta cell showed a compensatory adaptation to sub-chronic exposure to Aroclor 1254, which may be due to up-regulation of estrogen and androgen receptors; in contrast, the alpha cell did not show compensatory mechanisms and hence underwent the indicated mass decrease.

In the study by Santos-Silva et al., again in 2020 [77], the aim was to investigate the effects of in utero exposure to glucocorticoids (dexamethasone) on the development of the alpha cell (and beta cell) during postnatal life. Specifically, both morphological and transcriptional features of alpha and beta cells were analyzed. To this purpose, at 12 weeks of age, female Wistar rats were housed in individual cages with one male for three days, and when pregnant, the rats were isolated. On the 14th day of pregnancy, half of the pregnant rats (10 rats in total) started treatment with dexamethasone (0.1 mg/kg body weight) diluted in drinking water, until the 19th day of pregnancy. Daily liquid intake and body weight were monitored and used to adjust the concentration of dexamethasone necessary to provide the final dosage of 0.1 mg/kg/day, whereas the untreated pregnant rats were used as controls. It was found that rats born to dexamethasone-treated mothers had increased alpha-cell mass and circulating glucagon levels. Insulin levels were also increased, despite reduced beta-cell mass, which was explained by up-regulation in the expression of genes associated with insulin secretion. It was concluded that in utero exposure to glucocorticoids leads to postnatal changes in the morphology of the endocrine pancreas and to transcriptional changes in genes involved in the alpha- and beta-cell phenotypes.

A recent study (2022) by Asadi et al. [78] again addressed the issue of the effects of in utero exposure to compounds potentially harmful for glucose metabolism. The study moved from the consideration that the use of medical and recreational cannabis has been increasing worldwide in the last few years, and such use during pregnancy is a major concern for the possible adverse effect on the health of the offspring later in life. The aim of Asadi’s study was therefore to determine the effects of in utero exposure to delta-9-tetrahydrocannabinol (Δ9-THC) on the offspring’s alpha (and beta) cells and related glucagon (and insulin) patterns. To this purpose, pregnant Wistar rats were injected with Δ9-THC (3 mg/kg per day, intraperitoneally) or vehicle from gestational days 6 to 22 (birth), this injection regimen resulting in maternal blood Δ9-THC levels comparable to blood levels observed in humans who are moderate recreational cannabis users. The studied offspring was composed by four males and six females in each of the two groups (Δ9-THC and controls). In adult female offspring of the Δ9-THC group, an increased serum insulin-to-glucagon ratio was observed, though Δ9-THC did not alter fasting blood glucose and serum insulin levels in either male or female adult offspring. It was concluded that the increased insulin-to-glucagon ratio suggests an impairment in alpha-cell function and may have direct correlations with early glucose intolerance and insulin resistance, though in the studied prenatal cannabinoid model, the increased insulin-to-glucagon ratio alterations were observed exclusively in females’ offspring.

In another recent study, Morsi et al. [79] focused on the possible effects of bisphenol-A and dibutyl phthalate. In fact, humans are often exposed to such plasticizers, which are commonly present in plastic materials, in particular in products made of polyvinyl chloride. Specifically, bisphenol-A is a key chemical in polycarbonates, polystyrene, and epoxy resin production, whereas dibutyl phthalate is one of the famous phthalate esters that is contaminating the environment (microplastic pollution). The Morsi study hypothesized that both compounds may affect the alpha cells in albino rats when administered at environmentally relevant doses. Thus, 36 male Wistar albino rats were separated into four equal groups, undergoing administration of bisphenol-A, dibutyl phthalate, bisphenol-A and dibutyl phthalate combined, and none of the two (controls). Bisphenol-A and dibutyl phthalate were given in drinking water for 45 days at a dose of 4.5 and 0.8 µg/L, respectively. It was found that rats exposed to bisphenol-A alone show alpha-cell apoptosis only, whereas rats exposed to both chemicals show both alpha- and beta-cell apoptosis, as well as islet atrophy and reduced glucagon expression. In contrast, rats exposed to dibutyl phthalate alone showed no changes in either alpha or beta cells. In summary, those findings indicate that beta cells are somehow resistant to stress and, hence, to apoptosis, while alpha cells progress to apoptosis even when exposed to bisphenol-A alone. This suggests the interesting hypothesis that the recently called alpha to beta cell trans-differentiation in diabetic patients may be a bypass defense mechanism to skip alpha cell stress-induced apoptosis.

### 3.2. Fatty Acids-Derived Compounds

In our search of the scientific literature, we identified some studies that all focused on the possible effects on the alpha cell of compounds derived from fatty acids (especially palmitate, derived from palmitic acid). In fact, the peculiar interest in palmitate is likely due to its wide use as a low-cost additive in several industrial food products, determining a remarkable exposure of the general population to this compound. We grouped these studies on fatty acid-derived compounds in the present subsection.

In 2006, Hong et al. [80] observed that it was already well known (at that time) that long-term exposure to fatty acids impairs beta-cell function, whereas, in contrast, little was known about the possible effects on the alpha cell. Thus, the main aim of the study was to investigate the effects on alpha-cell function of chronic exposure to palmitate (a compound derived from palmitic acid by esterification). In addition, it was investigated whether stevioside (an antihyperglycemic agent) may be able to counteract the effects of palmitate. The experimental procedure was based on an in vitro analysis of alpha-tumor cell 1 clone 6 (alpha-TC1-6) cells derived from an adenoma in transgenic mice, which were cultured with palmitate in the presence or absence of stevioside. After 72 h, glucagon secretion, glucagon content, and changes in gene expression were assessed. It was observed that glucagon secretion increased in a dose-dependent manner, starting from a palmitate concentration of 0.25 mmol/L. In addition, from 0.5 mmol/L palmitate concentration onward, the triglyceride content of the alpha cells increased. As regards stevioside, at a concentration of 10^−6^ mol/L or even lower, it was able to reduce palmitate-stimulated glucagon release. It was concluded that prolonged exposure to high fatty acid levels leads to hypersecretion of glucagon and an accumulation of triglycerides in clonal alpha-TC1-6 cells, but stevioside can counteract the palmitate effects. Thus, such findings suggested that stevioside may be a promising antidiabetic agent for the treatment of type 2 diabetes, especially when associated with alpha-cell dysfunction and related hyperglucagonemia.

In 2008, Collins et al. [81] carried out a study similar to that by Hong et al. [80], but with some differences. Especially, pancreatic islets rather than alpha-TC1-6 were cultured, and in addition, the study focused not only on palmitate but also on oleate (an oleic acid-derived compound). Furthermore, oleate’s effects on somatostatin were also considered. The pancreatic islets (from NMRI mice) were cultured for 72 h, as in Hong’s study. The islet cultures were kept at 4.5 or 15 mmol/L glucose, with or without 0.5 mmol/L oleate or palmitate. The release of glucagon and somatostatin during subsequent 1 h incubation at 1 or 20 mmol/L glucose was determined, as well as the as well as the islet content of the two hormones. It was found that prolonged exposure to palmitate or oleate increased glucagon secretion at 1 mmol/L glucose by 50% (when islets had been cultured at 15 mmol/L glucose) to 100% (with 4.5 mmol/L glucose in the culture). In addition, palmitate or oleate essentially abolished the inhibitory effect of 20 mmol/L glucose on glucagon secretion. As regards somatostatin, glucose-induced somatostatin secretion was reduced by about 50% following prolonged exposure to either of the fatty acid compounds, either in 4.5 or 15 mmol/L glucose culture. It was concluded that prolonged exposure to high glucose and/or fatty acids affects the release of glucagon and somatostatin, and the specific effect on glucagon may be particularly remarkable.

The study by Kristinsson et al. in 2017 [82] hypothesized that palmitate simultaneously stimulates secretion of glucagon and insulin at fasting glucose concentrations, likely due to the free fatty acid receptor 1 (FFAR1/GPR40), which is present in alpha-cells. This hypothesis was suggested to Kristinsson et al. by previous findings, where it was shown that in human islets, palmitate is able to enhance glucose-stimulated insulin secretion via FFAR1. In the 2017 study, analyses were based on human islets obtained from brain-dead otherwise healthy individuals with no known metabolic diseases, as well as on the EndoC-βH1 cells (a cell line strongly resembling human beta cells in terms of glucose and incretin-stimulated insulin secretion abilities). It was found that the human islet’s basal glucagon and insulin secretion (but also somatostatin secretion) were increased during palmitate treatment at normoglycemia, but that secretion of all hormones was lowered when FFAR1 was inhibited. Some of those findings were confirmed in the EndoC-βH1 human beta-cell line. The main conclusion was that that fatty acids enhance both glucagon and insulin secretion at fasting glucose concentrations, but FFAR1 is crucial for these effects. From a clinical point of view, the ability of chronically elevated palmitate levels to simultaneously increase basal secretion of glucagon and insulin suggests that high levels of fatty acids can be triggering factors for the development of impaired glucose control and obesity.

The study by Filippello et al. in 2018 [83] is different from the others reported in previous paragraphs since it still focuses on glucagon secretion, but at the level of the intestinal L-cells rather than from the alpha cells. However, we nonetheless included it in our review study, since we hypothesize that some of the study findings may be relevant for the alpha cell as well. Indeed, it was reported that some observations suggested L-cells were able to process from proglucagon not only the incretin hormone glucagon-like peptide 1 (GLP-1), but also glucagon. The main aim of the Filippello study was therefore to investigate the effects on murine GLUTag L-cells of chronic palmitate exposure on glucagon secretion, in addition to GLP-1 secretion. To mimic lipotoxicity, cells were cultured in the presence of palmitate (0.5 mmol/L) or in the absence of palmitate for 24 h. It was found that the palmitate treatment increased proglucagon expression and glucagon secretion, likely due to up-regulation of prohormone convertase 2 (an enzyme responsible for the first steps in the maturation of many peptides from their precursors). In contrast, insulin-stimulated GLP-1 secretion was reduced. It was concluded that the study findings support the hypothesis of lipotoxicity as a contributor to L-cell dysregulation, with regard to both glucagon and GLP-1 secretion.

## 4. Physical Factors Effect on the Alpha Cell

In 1995, Oda et al. [84] performed a study investigating the effects of cold exposure on glucagon (as well as insulin) secretion in response to a variety of secretagogues; in addition, the effects of alpha- and beta-adrenergic blockade on the basal levels of plasma glucagon (and insulin) were evaluated. To achieve this aim, six mature non-lactating female goats of the Japanese Saanen breed were exposed to a warm environment (18–22 °C) and then to a cold environment (0 °C). In both environments, each goat underwent different stimulation experiments: glucose injection (0.625 mmol/kg); arginine injection (1.25 mmol/kg); butyrate injection (0.625 mmol/kg); tolbutamide injection (20 mg/kg); phentolamine infusion (alpha antagonist, 40 μg∙kg^−1^∙min^−1^ infused for 60 min); and propranolol infusion (beta antagonist, 60 μg∙kg^−1^∙min^−1^). Experiments were given every 3 days, and in the cold environment, they were started after 7 days of cold exposure. In all the experiments, blood samples were collected until 90 min from the injection/infusion onset for the measurement of glucagon (as well as insulin and glucose) concentration. The results of this study led to the conclusion that the glucagon responses to these stimuli did not differ between a cold and warm environment. On the other side, cold exposure significantly decreased the insulin response to arginine, butyrate, and tolbutamide secretagogues; a tendency to reduce the insulin response to glucose was observed. The effect of the alpha- and beta-adrenergic blockade on insulin secretion was found to be more effective in the cold than in a warm environment.

Afterwards, in the late 90s, Itoh and coworkers investigated the effects of heat exposure on plasma glucagon (as well as insulin and metabolites) in different animal species, using different stimulation protocols. In particular, in 1998, the first study by Itoh et al. [85] aimed to investigate this matter in heifers. To achieve this aim, four Holstein heifers were exposed first to a thermoneutral (temperature: 20 °C, relative humidity: 60%) and then to a hot (temperature: 30 °C, relative humidity: 60%) environment for a 30-day and 16-day period, respectively. In each environment, all the heifers underwent (on different days) four experiments consisting on the injection of the following test agents: glucose (0.625 mmol/kg), arginine (0.625 mmol/kg), butyrate (0.625 mmol/kg), and insulin (0.2 U/kg). Venous blood samples were collected until 180 min after the insulin injection and until 120 min for all the other experiments. From each blood sample, plasma glucagon (as well as insulin and metabolites) was measured. It was found that in the hot environment, glucagon secretion was significantly augmented with respect to the thermoneutral environment, especially in the arginine and butyrate experiments; in contrast, insulin secretion in response to butyrate was reduced. These observations led to the conclusion that in the hot environment, glucagon was more sensitive to secretagogues than insulin.

In the same year, another study by Itoh et al. [86] aimed to investigate the same matter in lactating cows. To this aim, four Holstein multiparous cows in midlactation were exposed first to a thermoneutral (temperature: 18 °C, relative humidity: 60%) and then to a hot (temperature: 28 °C, relative humidity: 60%) environment for a 17-day and 13-day period, respectively. In each environment, all the cows underwent (on different days) treatments with three different secretagogues, namely: a glucose injection (0.625 mmol/kg) followed by a 30-min infusion of a second dose of glucose (0.022 mmol∙kg^−1^∙min^−1^); an arginine injection (0.625 mmol/kg); a butyrate infusion (0.021 mmol∙kg^−1^∙min^−1^ over 30 min). Venous blood samples were collected until 180 min after the onset of the glucose and butyrate infusions and 120 min following the arginine injection. From each blood sample, plasma glucagon (as well as insulin, glucose, and non-esterified fatty acids) was measured. It was found that glucagon’s (as well as insulin) response to the glucose infusion was not affected by heat exposure. In response to the arginine injection, peak values of glucagon (as well as insulin) were significantly higher in the hot environment than in the thermoneutral environment; concomitantly, the increase in plasma glucose concentration was lower in the hot environment than in the thermoneutral environment. Finally, in response to butyrate infusion, the insulin peak value was higher in the hot environment. Since glucagon secretion in response to the arginine injection was increased by heat exposure but the plasma glucose increase was inhibited, it was concluded that it is likely that gluconeogenesis was diminished because of the reduced substrate supply and the reduced hepatic sensitivity to glucagon.

The study by Messias de Bragança and Prunier in 1999 [87] investigated the effects of ambient temperature together with feed level on plasma profiles of glucagon as well as glucose, nonesterified fatty acids, and insulin in primiparous lactating sows. Indeed, lactating sows, especially when primiparous, have high energy requirements for maintenance and milk production; on the other side, exposure to high ambient temperatures leads to a reduction in feed intake, which results in decreased milk production. The research hypothesis was that nutrient supply to the mammary glands, which depends on blood nutrient concentrations (with glucagon and insulin as main regulators), is affected by hot ambient temperatures. In this study, two replicates of 12 sows (Piétrain × Large White) were allocated to three different treatment groups during lactation based on environment temperature (i.e., thermoneutral: 20 °C; hot: 30 °C) and feeding regimen (restricted or ad libitum). Specifically, the first and second groups were placed in a thermoneutral environment with either ad libitum or restricted feeding (three sows per replicate, each); the third group was placed in a hot environment with ad libitum feeding (six sows per replicate). Serial blood samples were collected during lactation and postweaning (two days, from 60 min before to 180 min after the afternoon meal). Glucagon (in addition to insulin) was measured in serial blood samples. Results of the study showed that restricted feeding in lactating sows exposed to a thermoneutral environment induced increase in plasma glucagon, which favors glucose availability. In the conditions of ad libitum feeding, no significant increase was observed in glucagon before and after the meal, comparing sows in the hot environment with those in the thermoneutral environment. It was then concluded that this may possibly cause negative effects on glucose availability to the mammary gland and on milk production.

In 2000, the study by Morales et al. [88] aimed to investigate glucagon receptor gene expression and its modulation by sympathetic nerve activity in brown adipose tissue (BAT) during cold exposure. The research hypothesis originated from the knowledge that BAT is involved in cold-induced thermogenesis and that it is mainly controlled by sympathetic innervation. On the other side, it is also known that specific glucagon receptors are located on the membranes of brown adipocytes. In this study, a group of male Wistar rats underwent surgical sympathetic denervation of interscapular BAT (unilaterally), and four days after the surgery, rats were either exposed at 4 °C (cold-exposed rats) or kept at 25 °C (thermoneutral control rats) for one week. After animal sacrifice, both pads of interscapular BAT were dissected for total RNA extraction, and then a semi-quantitative reverse transcriptase-polymerase chain reaction (RT-PCR) assay was performed. Results of this study showed that cold exposure resulted in down-regulation of glucagon receptor gene expression (–44% in terms of the relative abundance of BAT glucagon receptor mRNA for cold-exposed rats with respect to thermoneutral control rats), but this effect was mediated by sympathetic nerve activity. Indeed, the cold-exposure effect was completely eliminated by previous surgical sympathetic denervation of interscapular BAT. These observations led to the conclusion that glucagon is not supposed to have a major role in the thermogenic control of BAT.

In 2001, another study by Itoh et al. [89] investigated the effects of heat exposure on pancreatic secretion of glucagon (as well as insulin) and the role of adrenergic modulation. To achieve this aim, five mature, nonpregnant, and nonlactating Suffolk ewes, fed at a maintenance level, were studied. Ewes were housed first in a thermoneutral environment (temperature 20 °C, relative humidity 70%) and then in a hot environment (temperature 30 °C, relative humidity 70%) for a 32-day and 24-day period, respectively; they underwent on different days three experimental protocols with administration in randomized order of saline, phentolamine (an alpha antagonist, 10 nmol/kg/min), and propranolol (a beta antagonist, 20 nmol/kg/min) over 75 min. In addition, epinephrine (1.0 nmol/kg/min) was also infused, starting from 15 min after the onset of saline, phentolamine, or propranolol and lasting for 60 min. The three experiments were repeated in both the thermoneutral and hot environments. Venous blood samples were collected 15 min before the infusion onset and then every 15 min after the infusion onset for a total of 120 min. Blood samples were analyzed for the assessment of plasma glucagon concentrations. It was found that basal glucagon was higher during heat exposure, although not significantly; moreover, in the hot environment, no effect of either alpha or beta-antagonists on glucagon secretion was observed, leading to the conclusion that the adrenergic modulation probably does not affect to a significant extent glucagon secretion during heat exposure.

## 5. Discussion

In this review study, we analyzed the current evidence regarding the effects of chemical, biological, and physical environmental factors on the alpha cell. Interestingly, we found studies focusing on the effects of different categories of compounds, including air pollutants, compounds of known toxicity present in everyday use objects, pharmacological agents, and compounds often present in food. In addition, some studies focused on the effects of physical factors, mainly heat exposure. However, despite the broad span of the investigated compound categories, the overall number of studies in the field has to be considered limited, especially in comparison to studies related to the effects of exposure to environmental factors on other relevant actors in glucose metabolism, such as insulin sensitivity [48,49,50,51,52,53,54,55,56,57,58,59,60,61] and beta-cell function [62,63,64,65,66,67,68,69,70,71,72]. Thus, questions arise about the reasons for such a small number of studies as compared to those on insulin action and the beta cell. We hypothesize that this may be due to an underestimation of the role of the alpha cell in the maintenance of glucose homeostasis. Indeed, in principle, the role of the alpha cell in the regulation of glycemic levels through glucagon-stimulated hepatic glucose production has been known for a long time, but in our opinion, only in recent years has the actual relevance of glucagon (and of the alpha cell in general) been more clearly elucidated. First, it has been shown that the effects of glucagon on glucose homeostasis are not limited to the stimulation of hepatic glucose production. In fact, other important effects have been suggested, such as glucagon regulation of energy balance and body fat mass through effects on energy expenditure and food intake, as well as effects on gastric emptying and amino acid metabolism [90,91,92,93,94,95], the latter having potential effects on beta-cell function (though partly still controversial [96,97,98]). Furthermore, it has been reported that glucagon has a direct potentiating effect on insulin secretion, somehow similar to the well-known effect of GLP-1, with whom glucagon shares its precursor [99]. On the other hand, it is becoming evident that insulin control on glucagon secretion and kinetics may be even more marked than glucose control [100,101], and several other factors have also been suggested as modulators of glucagon secretion, such as insulin-like growth factor-1, apolipoprotein A-1, and HDL cholesterol [102,103]. In addition, it is now clear that the alpha cell exerts effects on the maintenance of glucose homeostasis that are beyond the effects related to glucagon. As an example, acetylcholine is secreted by the alpha cells and appears to play a role in beta-cell function [99,104]. In summary, the reported considerations indicate that the crucial role played by the alpha cell in glucose homeostasis is now clearly established, and this motivated us to carry out this review study on the effects of the exposure of the alpha cell to different environmental factors. Of note, the high number of citations per year of some of the analyzed articles confirms the relevance of the environment and alpha cell issues.

In the methodological section of this review, we stated that we discarded the articles published before the nineties. This choice was based on the hypothesis that such early studies in the field were likely based on methodologies (including measurement procedures) remarkably different from those available for the more recent studies, thus including the latter in the review may have resulted somehow in a potential bias when reporting the main findings. In addition, it has to be noted that all those early articles dealt with the effects on the alpha cell of environmental temperature variations (heat or cold exposure, acute or prolonged) [105,106,107,108,109,110,111,112,113,114,115], and these issues were also addressed in the more recent studies that were included in our review (though it is worth noting that the latest studies on this issue are from the early 2000s).

Another aspect needs to be discussed. Indeed, the reader may wonder the reason why it should be important to analyze the environmental effects on specific physiologic factors involved in glucose homeostasis, such as in fact the beta or alpha-cell function. In other words, one may be doubtful about the actual clinical relevance of determining the effect of a specific environmental factor over a specific physiological factor. However, we believe that the potential relevance is remarkable, at least considered in perspective, for the near future. Indeed, there has recently been a vigorous move towards precision medicine in diabetes, including precision diagnostics [116,117,118,119,120,121,122]. Thus, we expect that soon it may become part of the clinical routine for assessing the specific metabolic defects of a patient with diabetes, or even simple prediabetes or dysglycemia. Indeed, in a patient, the main defect may be in the field of insulin resistance, beta-cell dysfunction, or in fact alpha-cell dysfunction (or other defects either): the goal of precision medicine will be identifying the most relevant personal defects for each patient and prescribing tailored strategies for the disease care or for diseased prevention. In such a context, we expect that among several other relevant pieces of information, knowledge of the harmful environmental exposures for the alpha cell, as well as for the beta cell or other physiological aspects, will certainly play an important role. As an example, in the event that one patient will be determined with a remarkable defect in alpha-cell function, appropriate strategies will be suggested to focus specifically on that aspect, possibly including limitation of the exposure to potentially alpha-cell harmful compounds for professional reasons. This appears also in line with the concept recently emerging in the “Total Worker Health” approach [123,124,125,126], where the occupational doctor can cooperate with the general practitioner, the medical specialist, and possibly other health professionals to ensure improved health care for the individual.

Some readers may also be interested in the cellular and molecular mechanisms involved in the described effects of chemical and ambient factors on the alpha cell. With regard to that, the majority of the studies emphasize the need for further investigations to elucidate such mechanisms clearly. Nonetheless, some possible mechanisms are proposed and discussed. In the study by Fang et al. [76], it was postulated that the decreased alpha-cell mass due to exposure to Aroclor 1254 was related to down-regulated expression of the aristaless-related homeobox (ARX), which is a protein encoded by the homonymous gene. As regards the studies focusing on in utero exposures, in the study by Santos-Silva et al. [77], it was reported that the observed changes in the morphology of the alpha cell due to dexamethasone exposure were paralleled by a persistent increase in proglucagon gene (GCG) protein expression. In the study by Asadi et al. [78], the reduction in glucagon secretion by the alpha cells due to in utero exposure to delta-9-tetrahydrocannabinol was linked to its ability to affect the development of the fetal pancreas. In fact, delta-9-tetrahydrocannabinol interacts with the cannabinoid receptor 1 (CB1R) and the transient receptor potential cation channel subfamily V member 1 (TRPV1), which on their own may act on the neuronal protein stathmin-2 (Stmn2), known to be a potent suppressor of glucagon secretion. In the study by Morsi et al. [79], which focused on the effects of bisphenol-A and dibutyl phthalate on the alpha cell, it was reported that exposure to those compounds determines pancreatic oxidative stress. This was denoted by an imbalance between malondialdehyde (MDA, a final product of lipid peroxidation often assumed as marker of oxidative stress) and superoxide dismutases (SOD, an enzyme acting as an important antioxidant defense against oxidative stress). This may be the trigger for the action of heat shock protein 60 (HSP60, a mitochondrial chaperone that elicits a heat shock response on exposure to cell stress in the attempt to assist protein folding and repair the damage). On its side, under certain conditions, HSP60 interacts with the caspase-3 protein, which may determine alpha-cell vulnerability to bisphenol-A/dibutyl phthalate-induced apoptotic changes. In summary, the variety of mechanisms reported in the above-mentioned studies indicates the complexity of the possible effects of the chemical compounds on the alpha cell. In contrast, in the study by Stošić et al. [75], no mechanisms were proposed for the effects of acrylamide on the alpha cell, though it appears quite evident that such effects are secondary to a general remodeling of the islets of Langerhans, especially a mass reduction of the beta cells. With regard to the mechanisms for the effects on the alpha cell of the fatty acid-derived compounds, the study by Hong et al. [80] suggested that the exaggerated glucagon secretion caused by palmitate is counterbalanced by stevioside through the enhanced expression of carnitine palmitoyltransferase I (CPT I, a mitochondrial enzyme), stearoyl-CoA desaturase (SCD, an endoplasmic reticulum enzyme), and peroxisome proliferator-activated receptor-γ (PPAR γ, a nuclear receptor). In the study by Collins et al. [81], it was postulated that some metabolites of the free fatty acids, like palmitoyl-coenzyme A, are able to activate the K_ATP_-channels, and this may contribute to the loss of glucose regulation of glucagon secretion. The study by Kristinsson et al. [82] suggested that the palmitate-induced hypersecretion of glucagon (and of insulin as well) is mediated by the action of the FFAR1/GPR40 receptor, which signals via phospholipase C (PLC), diacylglycerol (DAG), and protein kinase C and D1 (PKC/PKD1). This leads to enhanced Ca^2+^ mobilization from intracellular Ca^2+^ stores, activation of calcium channels, enhanced islet respiration, and ATP production, which eventually facilitates higher glucagon and the insulin secretion. The study by Filippello et al. [83] suggested that the palmitate-induced glucagon hypersecretion may be due to an unbalance in the ratio between two isoforms of insulin receptor, i.e., isoform A and isoform B. This led to increased paired box 6 protein (PAX6) and expression of proglucagon, especially prohormone convertase 2 (PC2), with the final effect of these phenomena being, in fact, a rise in glucagon secretion. In the present review study, we also analyzed the effects of physical environmental factors on the alpha cell. With regard to the possible mechanisms involved, in the study by Oda et al. [84], it was suggested that the sympatho-adrenomedullary system may play a role in determining the suppressed insulin response to stimuli in a cold environment; on the other side, no mechanism was advocated to explain the unaltered glucagon response, even though an alpha-adrenergic mechanism may be involved. Activation of alpha-adrenergic receptors was also mentioned as a possible mechanism in the first 1998 study by Itoh et al. [85] to explain the enhanced glucagon secretion following arginine (or butyrate) injection in the hot environment. Moreover, the alteration of the direct sensitivity of the pancreas to secretagogues in the blood, due to enhanced sympatho-adrenal activity, was believed to play a role. In the other study of the same year by the same authors [86], although similar results were found in terms of response to the arginine injection, no mechanism was postulated; different results were obtained in terms of glucagon in response to butyrate, but no mechanism was postulated as well. In the study by Messias de Bragança et al. [87], again no mechanism was advocated to explain, in the condition of ad libitum feeding, the non-significant increase in glucagon observed in the hot environment before and after the meal. However, considering low-level feeding, a variation in the amount of carbohydrates absorbed during the meal may explain the observed decrease in the insulin-to-glucagon ratio between a thermoneutral and hot environment. In the study by Morales et al. [88], it was supposed that the down-regulation of glucagon receptor gene expression due to cold exposure may be controlled by noradrenaline, and the molecular mechanisms involved in this process could involve intracellular cyclic adenosine monophosphate (cAMP). Eventually, results of the 2001 study by Itoh et al. [89] excluded the adrenergic nervous system as the responsible mechanism of glucagon secretion modification during heat exposure, but no alternative mechanisms were hypothesized. We can conclude that, despite the relative paucity of studies on the chemical and ambient factors affecting the alpha cell, the heterogeneity and complexity of the relevant mechanisms have already emerged clearly. This suggests that a remarkable scientific effort is still needed to investigate such mechanisms in depth.

## 6. Conclusions

In conclusion, in the present review study, for the first time, the current evidence about the effects of exposure to different environmental factors on the alpha cell was examined. Such factors included different categories of compounds, such as air pollutants, toxic compounds often present in everyday objects, pharmacological agents, food compounds, and extreme ambient temperatures. In the light of the relevant role of the alpha cell in glucose homeostasis that has clearly emerged in recent years and is consistent with the indications of precision medicine for diabetes care, we expect further studies related to the relationships between environmental exposures and alpha-cell function in the future.

## Figures and Tables

**Table 1:** Summary information related to the studies concerning the effects on the alpha cell of various chemical/biological compounds. The number of citations (total/per year) is from SCOPUS (last checked: 30 September 2022).

Reference	Main Aims	Type of Experiment/Population	Main Findings	Number of Article Citations
Stošić et al., 2018 [75]	Effect of acrylamide * on alpha-cell structural changes	30 male Wistar rats	Sub-chronic acrylamide treatment of rats leads to islet of Langerhans remodeling due to alpha-cell expansion	11/2.75
Fang et al., 2020 [76]	Effect of Aroclor 1254 * (polychlorinated biphenyl mixture) on alpha-cell mass	40 male mice	Sub-chronic Aroclor 1254 exposure leads to alpha-cell increased apoptosis and decreased neogenesis, thus overall resulting in decreased alpha-cell mass	6/3
Santos-Silva et al., 2020 [77]	Effect of in utero exposure to dexamethasone * on alpha-cell mass and glucagon levels in the offspring	10 female Wistar rats and related offspring	In utero exposure to dexamethasone leads to postnatal changes in the morphology of the alpha cell and to transcriptional changes in genes involved in alpha- and beta-cell phenotypes	7/3.5
Asadi et al., 2022 [78]	Effect of in utero exposure to delta-9-tetrahydrocannabinol ^†^ on glucagon and insulin levels in the offspring	20 pups (offspring: eight males, 12 females) from Wistar rats	In utero exposure to delta-9-tetrahydrocannabinol determines an increased insulin-to-glucagon ratio, suggesting impairment in alpha-cell function	0/0
Morsi et al., 2022 [79]	Effect of bisphenol-A * and dibutyl phthalate * on alpha-cell apoptosis	36 Wistar albino rats	Exposure to bisphenol-A determines alpha-cell apoptosis; exposure to bisphenol-A and dibutyl phthalate combined determine both alpha- and beta-cell apoptosis, islet atrophy, and reduced glucagon	0/0

* chemical (non-biological) compound; ^†^ biological compound.

**Table 2:** Summary information related to the studies concerning the effects on the alpha cell of fatty acids. The number of citations (total/per year) is from SCOPUS (last checked: 30 September 2022).

Reference	Main Aims	Type of Experiment/Population	Main Findings	Number of Article Citations
Hong et al., 2006 [80]	Effect of palmitate ^†^ on alpha-cell function and the counteracting effect of stevioside	Culture of clonal alpha-TC1-6 cells from transgenic mice	Prolonged exposure to high fatty acid levels leads to glucagon hypersecretion and triglyceride accumulation, but stevioside counteracts the fatty acid effects	27/1.69
Collins et al., 2008 [81]	Effect of palmitate and oleate ^†^ on glucagon and somatostatin secretion at different glucose levels	Culture of pancreatic islets from NMRI mice	Prolonged exposure to high glucose and/or fatty acids affects glucagon and somatostatin secretion, with larger effects on the former	24/1.71
Kristinsson et al., 2017 [82]	Effect of palmitate on glucagon and insulin secretion at basal glucose levels	Culture of human pancreatic islets (deceased donors), and EndoC-βH1 beta-cells	Fatty acid increase both glucagon and insulin secretion at fasting glucose concentrations, but the role of free fatty acid receptor 1 (FFAR1/GPR40) is crucial for these effects	33/6.6
Filippello et al., 2018 [83]	Effect of palmitate on glucagon and GLP-1 secretion of intestinal L-cells	Culture of murine GLUTag L-cells	Prolonged fatty acid exposure increases proglucagon expression and glucagon secretion due to the up-regulation of prohormone convertase 2	16/4

^†^ biological compound.

**Table 3:** Summary information related to the studies concerning the effects on the alpha cell of physical environmental factors (heat or cold exposure). The number of citations (total/per year) is from SCOPUS (last checked: 30 September 2022).

Reference	Main Aims	Type of Experiment/Population	Main Findings	Number of Article Citations
Oda et al., 1995 [84]	Effect of cold exposure on glucagon secretion in response to different secretagogues	Six mature non-lactating goats of Japanese Saanen breed	Glucagon responses to different secretagogues do not differ between cold and warm environment	0/0
Itoh et al., 1998 [85]	Effect of heat exposure on glucagon secretion in response to different stimulation protocols	Four Holstein heifers	In the hot environment, glucagon secretion is increased with respect to the thermoneutral environment, especially under arginine and butyrate stimulation	8/0.33
Itoh et al., 1998 (2nd article) [86]	Effect of heat exposure on glucagon secretion in response to different stimulation protocols	Four Holstein multiparous lactating cows	In the hot environment, glucagon secretion is increased with respect to the thermoneutral environment, especially under arginine	67/2.79
Messias de Bragança et al., 1999 [87]	Effect of ambient temperature together with feed level on plasma profiles of glucagon	12 + 12 (two replicates) primiparous lactating sows	Restricted feeding in sows exposed to a thermoneutral environment induces an increase in plasma glucagon, which favors glucose availability, whereas under ad libitum feeding no increase is observed in glucagon	28/1.21
Morales at al., 2000 [88]	Glucagon receptor gene expression and its modulation by sympathetic nerve activity in brown adipose tissue during cold exposure	Male Wistar rats (number not specified)	Cold exposure determines down-regulation of glucagon receptor gene expression, but the effect is mediated by sympathetic nerve activity	7/0.32
Itoh et al., 2001 [89]	Effect of heat exposure on glucagon secretion and the role of adrenergic modulation	Five mature nonpregnant and nonlactating Suffolk ewes	The adrenergic modulation does not affect glucagon secretion to a significant extent during heat exposure	7/0.33

## Data Availability

Not applicable.

## References

[1] Laiho J.E., Laitinen O.H., Malkamäki J., Puustinen L., Sinkkonen A., Pärkkä J., Hyöty H. (2022). Exposomic Determinants of Immune-Mediated Diseases: Special Focus on Type 1 Diabetes, Celiac Disease, Asthma, and Allergies: The HEDIMED Project Approach. Environ. Epidemiol..

[2] Zorena K., Michalska M., Kurpas M., Jaskulak M., Murawska A., Rostami S. (2022). Environmental Factors and the Risk of Developing Type 1 Diabetes-Old Disease and New Data. Biology.

[3] Houeiss P., Luce S., Boitard C. (2022). Environmental Triggering of Type 1 Diabetes Autoimmunity. Front. Endocrinol..

[4] Quinn L.M., Wong F.S., Narendran P. (2021). Environmental Determinants of Type 1 Diabetes: From Association to Proving Causality. Front. Immunol..

[5] Giwa A.M., Ahmed R., Omidian Z., Majety N., Karakus K.E., Omer S.M., Donner T., Hamad A.R.A. (2020). Current Understandings of the Pathogenesis of Type 1 Diabetes: Genetics to Environment. World J. Diabetes.

[6] Dedrick S., Sundaresh B., Huang Q., Brady C., Yoo T., Cronin C., Rudnicki C., Flood M., Momeni B., Ludvigsson J. (2020). The Role of Gut Microbiota and Environmental Factors in Type 1 Diabetes Pathogenesis. Front. Endocrinol..

[7] Butalia S., Kaplan G.G., Khokhar B., Haubrich S., Rabi D.M. (2020). The Challenges of Identifying Environmental Determinants of Type 1 Diabetes: In Search of the Holy Grail. Diabetes Metab. Syndr. Obes..

[8] Blanter M., Sork H., Tuomela S., Flodström-Tullberg M. (2019). Genetic and Environmental Interaction in Type 1 Diabetes: A Relationship Between Genetic Risk Alleles and Molecular Traits of Enterovirus Infection?. Curr. Diabetes Rep..

[9] Howard S.G. (2019). Exposure to Environmental Chemicals and Type 1 Diabetes: An Update. J. Epidemiol. Community Health.

[10] Xia Y., Xie Z., Huang G., Zhou Z. (2019). Incidence and Trend of Type 1 Diabetes and the Underlying Environmental Determinants. Diabetes Metab. Res. Rev..

[11] Esposito S., Toni G., Tascini G., Santi E., Berioli M.G., Principi N. (2019). Environmental Factors Associated With Type 1 Diabetes. Front. Endocrinol..

[12] Howard S.G. (2018). Developmental Exposure to Endocrine Disrupting Chemicals and Type 1 Diabetes Mellitus. Front. Endocrinol..

[13] Chia J.S.J., McRae J.L., Kukuljan S., Woodford K., Elliott R.B., Swinburn B., Dwyer K.M. (2017). A1 Beta-Casein Milk Protein and Other Environmental Pre-Disposing Factors for Type 1 Diabetes. Nutr. Diabetes.

[14] Hu Y., Wong F.S., Wen L. (2017). Antibiotics, Gut Microbiota, Environment in Early Life and Type 1 Diabetes. Pharmacol. Res..

[15] Miller K.M., Hart P.H., de Klerk N.H., Davis E.A., Lucas R.M. (2017). Are Low Sun Exposure and/or Vitamin D Risk Factors for Type 1 Diabetes?. Photochem. Photobiol. Sci..

[16] Beulens J.W.J., Pinho M.G.M., Abreu T.C., den Braver N.R., Lam T.M., Huss A., Vlaanderen J., Sonnenschein T., Siddiqui N.Z., Yuan Z. (2022). Environmental Risk Factors of Type 2 Diabetes-an Exposome Approach. Diabetologia.

[17] Roth K., Petriello M.C. (2022). Exposure to Per- and Polyfluoroalkyl Substances (PFAS) and Type 2 Diabetes Risk. Front. Endocrinol..

[18] Ghorbani Nejad B., Raeisi T., Janmohammadi P., Mehravar F., Zarei M., Dehghani A., Bahrampour N., Darijani M.H., Ahmadipour F., Mohajeri M. (2022). Mercury Exposure and Risk of Type 2 Diabetes: A Systematic Review and Meta-Analysis. Int. J. Clin. Pract..

[19] Zhou J., Lin Y., Liu Y., Chen K. (2021). Antibiotic Exposure and Risk of Type 2 Diabetes Mellitus: A Systematic Review and Meta-Analysis. Environ. Sci. Pollut. Res. Int..

[20] Vinceti M., Filippini T., Wise L.A., Rothman K.J. (2021). A Systematic Review and Dose-Response Meta-Analysis of Exposure to Environmental Selenium and the Risk of Type 2 Diabetes in Nonexperimental Studies. Environ. Res..

[21] Gang N., Van Allen K., Villeneuve P.J., MacDonald H., Bruin J.E. (2021). Sex-Specific Associations between Type 2 Diabetes Incidence and Exposure to Dioxin and Dioxin-like Pollutants: A Meta-Analysis. Front. Toxicol..

[22] Bailey M.J., Naik N.N., Wild L.E., Patterson W.B., Alderete T.L. (2020). Exposure to Air Pollutants and the Gut Microbiota: A Potential Link between Exposure, Obesity, and Type 2 Diabetes. Gut Microbes.

[23] Misra B.B., Misra A. (2020). The Chemical Exposome of Type 2 Diabetes Mellitus: Opportunities and Challenges in the Omics Era. Diabetes Metab. Syndr..

[24] Yang M., Cheng H., Shen C., Liu J., Zhang H., Cao J., Ding R. (2020). Effects of Long-Term Exposure to Air Pollution on the Incidence of Type 2 Diabetes Mellitus: A Meta-Analysis of Cohort Studies. Environ. Sci. Pollut. Res. Int..

[25] Zafarmand M.H., Tajik P., Spijker R., Agyemang C. (2020). Gene-Environment Interaction on the Risk of Type 2 Diabetes Among Ethnic Minority Populations Living in Europe and North America: A Systematic Review. Curr. Diabetes Rev..

[26] Liu Y., Lou X. (2020). Type 2 Diabetes Mellitus-Related Environmental Factors and the Gut Microbiota: Emerging Evidence and Challenges. Clinics.

[27] Geng T., Huang T. (2020). Gene-Environment Interactions and Type 2 Diabetes. Asia Pac. J. Clin. Nutr..

[28] Liu F., Chen G., Huo W., Wang C., Liu S., Li N., Mao S., Hou Y., Lu Y., Xiang H. (2019). Associations between Long-Term Exposure to Ambient Air Pollution and Risk of Type 2 Diabetes Mellitus: A Systematic Review and Meta-Analysis. Environ. Pollut..

[29] Kadayifci F.Z., Haggard S., Jeon S., Ranard K., Tao D., Pan Y.-X. (2019). Early-Life Programming of Type 2 Diabetes Mellitus: Understanding the Association between Epigenetics/Genetics and Environmental Factors. Curr. Genom..

[30] Li Y., Xu L., Shan Z., Teng W., Han C. (2019). Association between Air Pollution and Type 2 Diabetes: An Updated Review of the Literature. Ther. Adv. Endocrinol. Metab..

[31] Hwang S., Lim J.-E., Choi Y., Jee S.H. (2018). Bisphenol A Exposure and Type 2 Diabetes Mellitus Risk: A Meta-Analysis. BMC Endocr. Disord..

[32] Vinceti M., Filippini T., Rothman K.J. (2018). Selenium Exposure and the Risk of Type 2 Diabetes: A Systematic Review and Meta-Analysis. Eur. J. Epidemiol..

[33] Rienks J., Barbaresko J., Oluwagbemigun K., Schmid M., Nöthlings U. (2018). Polyphenol Exposure and Risk of Type 2 Diabetes: Dose-Response Meta-Analyses and Systematic Review of Prospective Cohort Studies. Am. J. Clin. Nutr..

[34] Dendup T., Feng X., Clingan S., Astell-Burt T. (2018). Environmental Risk Factors for Developing Type 2 Diabetes Mellitus: A Systematic Review. Int. J. Environ. Res. Public Health.

[35] Lee Y.-M., Jacobs D.R., Lee D.-H. (2018). Persistent Organic Pollutants and Type 2 Diabetes: A Critical Review of Review Articles. Front. Endocrinol..

[36] Bellou V., Belbasis L., Tzoulaki I., Evangelou E. (2018). Risk Factors for Type 2 Diabetes Mellitus: An Exposure-Wide Umbrella Review of Meta-Analyses. PLoS ONE.

[37] Zhou X., Li C., Cheng H., Xie J., Li F., Wang L., Ding R. (2022). Association between Ambient Air Pollution Exposure during Pregnancy and Gestational Diabetes Mellitus: A Meta-Analysis of Cohort Studies. Environ. Sci. Pollut. Res. Int..

[38] Eberle C., Stichling S. (2022). Environmental Health Influences in Pregnancy and Risk of Gestational Diabetes Mellitus: A Systematic Review. BMC Public Health.

[39] Birru R.L., Liang H.-W., Farooq F., Bedi M., Feghali M., Haggerty C.L., Mendez D.D., Catov J.M., Ng C.A., Adibi J.J. (2021). A Pathway Level Analysis of PFAS Exposure and Risk of Gestational Diabetes Mellitus. Environ. Health.

[40] Kunysz M., Mora-Janiszewska O., Darmochwał-Kolarz D. (2021). Epigenetic Modifications Associated with Exposure to Endocrine Disrupting Chemicals in Patients with Gestational Diabetes Mellitus. Int. J. Mol. Sci..

[41] Pace N.P., Vassallo J., Calleja-Agius J. (2021). Gestational Diabetes, Environmental Temperature and Climate Factors—From Epidemiological Evidence to Physiological Mechanisms. Early Hum. Dev..

[42] Lin Y., Li T., Xiao J., Xie K., Shi Z. (2021). The Association Between Cadmium Exposure and Gestational Diabetes Mellitus: A Systematic Review and Meta-Analysis. Front. Public Health.

[43] Salmeri N., Villanacci R., Ottolina J., Bartiromo L., Cavoretto P., Dolci C., Lembo R., Schimberni M., Valsecchi L., Viganò P. (2020). Maternal Arsenic Exposure and Gestational Diabetes: A Systematic Review and Meta-Analysis. Nutrients.

[44] Zhang H., Wang Q., He S., Wu K., Ren M., Dong H., Di J., Yu Z., Huang C. (2020). Ambient Air Pollution and Gestational Diabetes Mellitus: A Review of Evidence from Biological Mechanisms to Population Epidemiology. Sci. Total Environ..

[45] Tang X., Zhou J.-B., Luo F., Han Y., Heianza Y., Cardoso M.A., Qi L. (2020). Air Pollution and Gestational Diabetes Mellitus: Evidence from Cohort Studies. BMJ Open Diabetes Res. Care.

[46] Hu C.-Y., Gao X., Fang Y., Jiang W., Huang K., Hua X.-G., Yang X.-J., Chen H.-B., Jiang Z.-X., Zhang X.-J. (2020). Human Epidemiological Evidence about the Association between Air Pollution Exposure and Gestational Diabetes Mellitus: Systematic Review and Meta-Analysis. Environ. Res..

[47] Elshahidi M.H. (2019). Outdoor Air Pollution and Gestational Diabetes Mellitus: A Systematic Review and Meta-Analysis. Iran. J. Public Health.

[48] Gao H., Chen D., Zang M. (2021). Association between Phthalate Exposure and Insulin Resistance: A Systematic Review and Meta-Analysis Update. Environ. Sci. Pollut. Res. Int..

[49] Huang R. (2021). Gut Microbiota: A Key Regulator in the Effects of Environmental Hazards on Modulates Insulin Resistance. Front. Cell. Infect. Microbiol..

[50] Shoshtari-Yeganeh B., Zarean M., Mansourian M., Riahi R., Poursafa P., Teiri H., Rafiei N., Dehdashti B., Kelishadi R. (2019). Systematic Review and Meta-Analysis on the Association between Phthalates Exposure and Insulin Resistance. Environ. Sci. Pollut. Res. Int..

[51] Kim Y.A., Park J.B., Woo M.S., Lee S.Y., Kim H.Y., Yoo Y.H. (2019). Persistent Organic Pollutant-Mediated Insulin Resistance. Int. J. Environ. Res. Public Health.

[52] Dang J., Yang M., Zhang X., Ruan H., Qin G., Fu J., Shen Z., Tan A., Li R., Moore J. (2018). Associations of Exposure to Air Pollution with Insulin Resistance: A Systematic Review and Meta-Analysis. Int. J. Environ. Res. Public Health.

[53] Roy C., Tremblay P.-Y., Ayotte P. (2017). Is Mercury Exposure Causing Diabetes, Metabolic Syndrome and Insulin Resistance? A Systematic Review of the Literature. Environ. Res..

[54] Calderón-Garcidueñas L., de la Monte S.M. (2017). Apolipoprotein E4, Gender, Body Mass Index, Inflammation, Insulin Resistance, and Air Pollution Interactions: Recipe for Alzheimer’s Disease Development in Mexico City Young Females. J. Alzheimers Dis..

[55] Mostafalou S. (2016). Persistent Organic Pollutants and Concern Over the Link with Insulin Resistance Related Metabolic Diseases. Rev. Environ. Contam. Toxicol..

[56] Dunlop K., Cedrone M., Staples J.F., Regnault T.R.H. (2015). Altered Fetal Skeletal Muscle Nutrient Metabolism Following an Adverse in Utero Environment and the Modulation of Later Life Insulin Sensitivity. Nutrients.

[57] Hectors T.L.M., Vanparys C., Van Gaal L.F., Jorens P.G., Covaci A., Blust R. (2013). Insulin Resistance and Environmental Pollutants: Experimental Evidence and Future Perspectives. Environ. Health Perspect..

[58] Underwood P.C., Chamarthi B., Williams J.S., Sun B., Vaidya A., Raby B.A., Lasky-Su J., Hopkins P.N., Adler G.K., Williams G.H. (2012). Replication and Meta-Analysis of the Gene-Environment Interaction between Body Mass Index and the Interleukin-6 Promoter Polymorphism with Higher Insulin Resistance. Metabolism.

[59] Latini G., Marcovecchio M.L., Del Vecchio A., Gallo F., Bertino E., Chiarelli F. (2009). Influence of Environment on Insulin Sensitivity. Environ. Int..

[60] Ting J.W., Lautt W.W. (2006). The Effect of Acute, Chronic, and Prenatal Ethanol Exposure on Insulin Sensitivity. Pharmacol. Ther..

[61] Weiss E.P., Brown M.D., Shuldiner A.R., Hagberg J.M. (2002). Fatty Acid Binding Protein-2 Gene Variants and Insulin Resistance: Gene and Gene-Environment Interaction Effects. Physiol. Genom..

[62] Marković Filipović J., Karan J., Ivelja I., Matavulj M., Stošić M. (2022). Acrylamide and Potential Risk of Diabetes Mellitus: Effects on Human Population, Glucose Metabolism and Beta-Cell Toxicity. Int. J. Mol. Sci..

[63] Asahara S.-I., Inoue H., Kido Y. (2022). Regulation of Pancreatic β-Cell Mass by Gene-Environment Interaction. Diabetes Metab. J..

[64] Schumacher L., Abbott L.C. (2017). Effects of Methyl Mercury Exposure on Pancreatic Beta Cell Development and Function. J. Appl. Toxicol..

[65] Janikiewicz J., Hanzelka K., Kozinski K., Kolczynska K., Dobrzyn A. (2015). Islet β-Cell Failure in Type 2 Diabetes—Within the Network of Toxic Lipids. Biochem. Biophys. Res. Commun..

[66] Nielsen J.H., Haase T.N., Jaksch C., Nalla A., Søstrup B., Nalla A.A., Larsen L., Rasmussen M., Dalgaard L.T., Gaarn L.W. (2014). Impact of Fetal and Neonatal Environment on Beta Cell Function and Development of Diabetes. Acta Obstet. Gynecol. Scand..

[67] De Tata V. (2014). Association of Dioxin and Other Persistent Organic Pollutants (POPs) with Diabetes: Epidemiological Evidence and New Mechanisms of Beta Cell Dysfunction. Int. J. Mol. Sci..

[68] Hectors T.L.M., Vanparys C., van der Ven K., Martens G.A., Jorens P.G., Van Gaal L.F., Covaci A., De Coen W., Blust R. (2011). Environmental Pollutants and Type 2 Diabetes: A Review of Mechanisms that Can Disrupt Beta Cell Function. Diabetologia.

[69] Zraika S., Hull R.L., Verchere C.B., Clark A., Potter K.J., Fraser P.E., Raleigh D.P., Kahn S.E. (2010). Toxic Oligomers and Islet Beta Cell Death: Guilty by Association or Convicted by Circumstantial Evidence?. Diabetologia.

[70] Morgan N.G. (2009). Fatty Acids and Beta-Cell Toxicity. Curr. Opin. Clin. Nutr. Metab. Care.

[71] Roche E., Maestre I., Martín F., Fuentes E., Casero J., Reig J.A., Soria B. (2000). Nutrient Toxicity in Pancreatic Beta-Cell Dysfunction. J. Physiol. Biochem..

[72] Dahlquist G.G. (1997). Viruses and Other Perinatal Exposures as Initiating Events for Beta-Cell Destruction. Ann. Med..

[73] Defronzo R.A. (2009). Banting Lecture. From the Triumvirate to the Ominous Octet: A New Paradigm for the Treatment of Type 2 Diabetes Mellitus. Diabetes.

[74] Holter M.M., Saikia M., Cummings B.P. (2022). Alpha-Cell Paracrine Signaling in the Regulation of Beta-Cell Insulin Secretion. Front. Endocrinol..

[75] Stošić M., Matavulj M., Marković J. (2018). Subchronic Exposure to Acrylamide Leads to Pancreatic Islet Remodeling Determined by Alpha Cell Expansion and Beta Cell Mass Reduction in Adult Rats. Acta Histochem..

[76] Fang L., Zhang S., Ou K., Zuo Z., Yu A., Wang C. (2020). Exposure to Aroclor 1254 Differentially Affects the Survival of Pancreatic β-Cells and α-Cells in the Male Mice and the Potential Reason. Ecotoxicol. Environ. Saf..

[77] Santos-Silva J.C., da Silva P.M.R., de Souza D.N., Teixeira C.J., Bordin S., Anhê G.F. (2020). In Utero Exposure to Dexamethasone Programs the Development of the Pancreatic β- and α-Cells during Early Postnatal Life. Life Sci..

[78] Asadi F., Fernandez Andrade J.A., Gillies R., Lee K., Dhanvantari S., Hardy D.B., Arany E.J. (2022). Sex-Dependent Effect of In-Utero Exposure to Δ9-Tetrahydrocannabinol on Glucagon and Stathmin-2 in Adult Rat Offspring. Can. J. Diabetes.

[79] Morsi A.A., Mersal E.A., Alsabih A.O., Abdelmoneim A.M., Sakr E.M., Alakabawy S., Elfawal R.G., Naji M., Aljanfawe H.J., Alshateb F.H. (2022). Apoptotic Susceptibility of Pancreatic Alpha Cells to Environmentally Relevant Dose Levels of Bisphenol-A versus Dibutyl Phthalate Is Mediated by HSP60/Caspase-3 Expression in Male Albino Rats. Cell Biol. Int..

[80] Hong J., Chen L., Jeppesen P.B., Nordentoft I., Hermansen K. (2006). Stevioside Counteracts the α-Cell Hypersecretion Caused by Long-Term Palmitate Exposure. Am. J. Physiol. Endocrinol. Metab..

[81] Collins S.C., Salehi A., Eliasson L., Olofsson C.S., Rorsman P. (2008). Long-Term Exposure of Mouse Pancreatic Islets to Oleate or Palmitate Results in Reduced Glucose-Induced Somatostatin and Oversecretion of Glucagon. Diabetologia.

[82] Kristinsson H., Sargsyan E., Manell H., Smith D.M., Göpel S.O., Bergsten P. (2017). Basal Hypersecretion of Glucagon and Insulin from Palmitate-Exposed Human Islets Depends on FFAR1 but Not Decreased Somatostatin Secretion. Sci. Rep..

[83] Filippello A., Urbano F., Di Mauro S., Scamporrino A., Di Pino A., Scicali R., Rabuazzo A.M., Purrello F., Piro S. (2018). Chronic Exposure to Palmitate Impairs Insulin Signaling in an Intestinal L-Cell Line: A Possible Shift from GLP-1 to Glucagon Production. Int. J. Mol. Sci..

[84] Oda S., Ikuta M., Kuhara T., Ohneda A., Sasaki Y. (1995). Insulin and Glucagon Secretion in Goats (*Capra hircus* Linnæus) Exposed to Cold. Comp. Biochem. Physiol. Part C Pharmacol. Toxicol. Endocrinol..

[85] Itoh F., Obara Y., Fuse H., Rose M.T., Osaka I., Takahashi H. (1998). Effects of Heat Exposure on Plasma Insulin, Glucagon and Metabolites in Response to Nutrient Injection in Heifers. Comp. Biochem. Physiol. Part C Pharmacol. Toxicol. Endocrinol..

[86] Itoh F., Obara Y., Rose M.T., Fuse H., Hashimoto H. (1998). Insulin and Glucagon Secretion in Lactating Cows during Heat Exposure. J. Anim. Sci..

[87] de Bragança M.M., Prunier A. (1999). Effects of Low Feed Intake and Hot Environment on Plasma Profiles of Glucose, Nonesterified Fatty Acids, Insulin, Glucagon, and IGF-I in Lactating Sows. Domest. Anim. Endocrinol..

[88] Morales A., Lachuer J., Géoën A., Georges B., Duchamp C., Barré H. (2000). Sympathetic Control of Glucagon Receptor MRNA Levels in Brown Adipose Tissue of Cold-Exposed Rats. Mol. Cell. Biochem..

[89] Itoh F., Hodate K., Koyama S., Rose M.T., Matsumoto M., Ozawa A., Obara Y. (2001). Effects of Heat Exposure on Adrenergic Modulation of Insulin and Glucagon Secretion in Sheep. Endocr. J..

[90] (2010). Glucagon Regulation of Energy Metabolism. Physiol. Behav..

[91] Marroquí L., Alonso-Magdalena P., Merino B., Fuentes E., Nadal A., Quesada I. (2014). Nutrient Regulation of Glucagon Secretion: Involvement in Metabolism and Diabetes. Nutr. Res. Rev..

[92] Charron M.J., Vuguin P.M. (2015). Lack of Glucagon Receptor Signaling and Its Implications beyond Glucose Homeostasis. J. Endocrinol..

[93] Sandoval D.A., D’Alessio D.A. (2015). Physiology of Proglucagon Peptides: Role of Glucagon and GLP-1 in Health and Disease. Physiol. Rev..

[94] Ahrén B. (2015). Glucagon--Early Breakthroughs and Recent Discoveries. Peptides.

[95] Wewer Albrechtsen N.J., Kuhre R.E., Pedersen J., Knop F.K., Holst J.J. (2016). The Biology of Glucagon and the Consequences of Hyperglucagonemia. Biomark. Med..

[96] Ding C., Egli L., Bosco N., Sun L., Goh H.J., Yeo K.K., Yap J.J.L., Actis-Goretta L., Leow M.K.-S., Magkos F. (2021). Plasma Branched-Chain Amino Acids Are Associated With Greater Fasting and Postprandial Insulin Secretion in Non-Diabetic Chinese Adults. Front. Nutr..

[97] Karusheva Y., Strassburger K., Markgraf D.F., Zaharia O.-P., Bódis K., Kössler T., Tura A., Pacini G., Burkart V., Roden M. (2021). Branched-Chain Amino Acids Associate Negatively With Postprandial Insulin Secretion in Recent-Onset Diabetes. J. Endocr. Soc..

[98] Morettini M., Palumbo M., Göbl C., Burattini L., Karusheva Y., Roden M., Pacini G., Tura A. (2022). Mathematical Model of Insulin Kinetics Accounting for the Amino Acids Effect during a Mixed Meal Tolerance Test. Front. Endocrinol..

[99] Moede T., Leibiger I.B., Berggren P.-O. (2020). Alpha Cell Regulation of Beta Cell Function. Diabetologia.

[100] Morettini M., Burattini L., Göbl C., Pacini G., Ahrén B., Tura A. (2021). Mathematical Model of Glucagon Kinetics for the Assessment of Insulin-Mediated Glucagon Inhibition During an Oral Glucose Tolerance Test. Front. Endocrinol..

[101] Göbl C., Morettini M., Salvatori B., Alsalim W., Kahleova H., Ahrén B., Tura A. (2022). Temporal Patterns of Glucagon and Its Relationships with Glucose and Insulin Following Ingestion of Different Classes of Macronutrients. Nutrients.

[102] Mancuso E., Mannino G.C., Fatta C.D., Fuoco A., Spiga R., Andreozzi F., Sesti G. (2017). Insulin-like Growth Factor-1 Is a Negative Modulator of Glucagon Secretion. Oncotarget.

[103] Mancuso E., Mannino G.C., Fuoco A., Leo A., Citraro R., Averta C., Spiga R., Russo E., De Sarro G., Andreozzi F. (2020). HDL (High-Density Lipoprotein) and ApoA-1 (Apolipoprotein A-1) Potentially Modulate Pancreatic α-Cell Glucagon Secretion. Arterioscler. Thromb. Vasc. Biol..

[104] Huising M.O. (2020). Paracrine Regulation of Insulin Secretion. Diabetologia.

[105] Heim T., Hull D. (1966). The Effect of Propranalol on the Calorigenic Response in Brown Adipose Tissue of New-Born Rabbits to Catecholamines, Glucagon, Corticotrophin and Cold Exposure. J. Physiol..

[106] Fekete M., Milner R.D.G., Soltész G., Assan R., Mestyán J. (1972). Plasma Glucagon, Thyrotropin, Growth Hormone and Insulin Response to Cold Exposure in the Human Newborn. Acta Paediatr..

[107] Kuroshima A., Yahata T., Ohno T. (1981). Changes in Plasma Glucagon Levels to Stressful Environmental Temperatures. Jpn. J. Physiol..

[108] Seitz H.J., Krone W., Wilke H., Tarnowski W., Carsten D., Dunkelmann B., Harneit A. (1981). Rapid Rise in Plasma Glucagon Induced by Acute Cold Exposure in Man and Rat. Pflugers Arch..

[109] Sasaki Y., Takahashi H., Aso H., Ohneda A., Weekes T.E.C. (1982). Effects of Cold Exposure on Insulin and Glucagon Secretion in Sheep. Endocrinology.

[110] Takahashi H. (1984). Effects of Arginine and Cold Exposure on Secretory Responses of Insulin, Glucagon and 11-Hydroxycorticosteroids in Piglets. Nihon Juigaku Zasshi.

[111] Takahashi H., Murata H., Matsumoto H. (1985). Secretory Responses of Insulin, Glucagon and 11-Hydroxycorticosteroids to Arginine Injection in Calves Exposed to Heat. Nihon Juigaku Zasshi.

[112] Howland R.J. (1986). Acute Cold Exposure Increases the Glucagon Sensitivity of Thermogenic Metabolism in the Rat. Experientia.

[113] Takahashi H., Murata H., Matsumoto H. (1986). Responses of Plasma Insulin, Glucagon and Cortisol to Cold Exposure in Calves. Nihon Juigaku Zasshi.

[114] Takahashi H. (1986). Effects of Glucose Injection and Cold Exposure on Secretory Responses of Insulin, Glucagon and 11-Hydroxycorticosteroids in Piglets. Nihon Juigaku Zasshi.

[115] Takahashi H., Murata H., Matsumoto H. (1986). Secretory Responses of Plasma Insulin, Glucagon, Cortisol and Glucose to Heat Exposure in Calves. Nihon Juigaku Zasshi.

[116] Chung W.K., Erion K., Florez J.C., Hattersley A.T., Hivert M.-F., Lee C.G., McCarthy M.I., Nolan J.J., Norris J.M., Pearson E.R. (2020). Precision Medicine in Diabetes: A Consensus Report From the American Diabetes Association (ADA) and the European Association for the Study of Diabetes (EASD). Diabetes Care.

[117] Florez J.C., Pearson E.R. (2022). A Roadmap to Achieve Pharmacological Precision Medicine in Diabetes. Diabetologia.

[118] Herder C., Roden M. (2022). A Novel Diabetes Typology: Towards Precision Diabetology from Pathogenesis to Treatment. Diabetologia.

[119] Hulman A., Foreman Y.D., Brouwers M.C.G.J., Kroon A.A., Reesink K.D., Dagnelie P.C., van der Kallen C.J.H., van Greevenbroek M.M.J., Færch K., Vistisen D. (2021). Towards Precision Medicine in Diabetes? A Critical Review of Glucotypes. PLoS Biol..

[120] Misra S., Florez J.C. (2022). Extending Precision Medicine Tools to Populations at High Risk of Type 2 Diabetes. PLoS Med..

[121] Nolan J.J., Kahkoska A.R., Semnani-Azad Z., Hivert M.-F., Ji L., Mohan V., Eckel R.H., Philipson L.H., Rich S.S., Gruber C. (2022). ADA/EASD Precision Medicine in Diabetes Initiative: An International Perspective and Future Vision for Precision Medicine in Diabetes. Diabetes Care.

[122] Schiborn C., Schulze M.B. (2022). Precision Prognostics for the Development of Complications in Diabetes. Diabetologia.

[123] Bradley C.J., Grossman D.C., Hubbard R.A., Ortega A.N., Curry S.J. (2016). Integrated Interventions for Improving Total Worker Health: A Panel Report From the National Institutes of Health Pathways to Prevention Workshop: Total Worker Health-What’s Work Got to Do With It?. Ann. Intern. Med..

[124] Rogers B., Schill A.L. (2021). Ethics and Total Worker Health^®^: Constructs for Ethical Decision-Making and Competencies for Professional Practice. Int. J. Environ. Res. Public Health.

[125] Schill A.L., Chosewood L.C. (2013). The NIOSH Total Worker Health^TM^ Program: An Overview. J. Occup. Environ. Med..

[126] Tamers S.L., Chosewood L.C., Childress A., Hudson H., Nigam J., Chang C.-C. (2019). Total Worker Health^®^ 2014–2018: The Novel Approach to Worker Safety, Health, and Well-Being Evolves. Int. J. Environ. Res. Public Health.

